# Leg length change after opening wedge and closing wedge high tibial osteotomy: A meta-analysis

**DOI:** 10.1371/journal.pone.0181328

**Published:** 2017-07-13

**Authors:** Jun-Ho Kim, Hyun-Jung Kim, Dae-Hee Lee

**Affiliations:** 1 Department of Orthopaedic Surgery, Samsung Medical Center, Sungkyunkwan University School of Medicine, Seoul, Korea; 2 Department of Preventive medicine, Korea University College of Medicine, Seoul, Korea; Harvard Medical School/BIDMC, UNITED STATES

## Abstract

**Background:**

Theoretical considerations suggest that leg length increases after opening wedge high tibial osteotomy (HTO) and decreases after closing wedge HTO; however, in vivo studies have yielded conflicting results. This meta-analysis therefore assessed changes in leg length after opening wedge and closing wedge HTO.

**Methods:**

All studies comparing pre- and postoperative leg length in patients who underwent opening and/or closing wedge HTO were included. Two reviewers independently recorded data from each study in terms of sample size as well as preoperative and postoperative leg length of open wedge and/or closed wedge HTO groups.

**Results:**

Four studies were included in the meta-analysis. Although pooled results showed leg length changes from before to after surgery were –6.93 mm (95% confidence interval [CI]: –17.53 to 3.67 mm; P = 0.20) in opening wedge HTO and 1.97 mm (95% CI: –7.13 to 11.07 mm; P = 0.67) in closing wedge HTO, respectively, these values were statistically not significant. However, the difference in the pooled mean leg length change from before to after surgery between opening wedge and closing wedge HTO was 8 mm, a difference that was significant (95% CI: 6.53 to 9.46 mm; P<0.001).

**Conclusion:**

The change in leg length was not statistically significant for either opening or closing wedge HTO. However, leg length change from before to after surgery was 8 mm greater for opening wedge HTO than for closing wedge HTO.

## Introduction

High tibial osteotomy (HTO) is accepted as the established treatment for patients with early medial compartment knee osteoarthritis and varus deformity.[1] Among various types of HTO, the two most commonly used types are opening wedge and closing wedge HTO.[2] The rationale of the procedure for pain relief is to shift the weight bearing force from the arthritic medial compartment to the intact lateral compartment by correcting alignment in the coronal plane.[3–5] The consideration of alignment correction in only the coronal plane can lead to unintended effects such as a change in the posterior tibial slope,[6,7] patellar height,[8] and rotation of the distal tibia below the osteotomy site.[9,10] One of these unintended alterations is leg length change after opening or closing wedge HTO. Theoretically, leg length increases after opening wedge HTO due to spreading of the osteotomy site in the proximal tibia and decreases after closing wedge HTO due to bone loss by removal of the osteotomy area in the proximal tibia. However, this phenomenon in HTO has received relatively little attention from orthopedic surgeons; there is no general consensus on whether leg length is actually increased after opening wedge HTO and decreased after closing wedge HTO, and previous studies[11–14] showed conflicting results. In addition, it is unclear whether the magnitude of leg length changes after surgery is different between opening and closing wedge HTO.

This study was designed to verify whether leg length after surgery is actually increased in opening wedge HTO and decreased in closing wedge HTO, as well as to quantify the difference in leg length change following surgery between opening and closing wedge HTO. It was hypothesized that leg length actually increases after opening wedge HTO and decreases after closing wedge HTO, and that the amount of leg length changes are similar between opening and closing wedge HTO.

## Materials and methods

This meta-analysis was performed according to the guidelines of the preferred reporting items for systematic reviews and meta-analysis (PRISMA) statement (S1 PRISMA Checklist).

### Data & literature sources

The study design was based on Cochrane Review Methods. Multiple comprehensive databases, including MEDLINE (January 1, 1976 to May 31, 2015), EMBASE (January 1, 1985 to May 31, 2015), the Cochrane Library (January 1, 1987 to May 31, 2015) and KoreaMed (June 1, 1958 to May 31, 2015), were searched for studies that evaluated the leg length in patients who underwent open wedge and/or closed wedge HTO (S1 Search Strategy). There were no restrictions on language or year of publication. Search terms used in the title, abstract, MeSH and keyword fields included "Osteotomy" [tiab] OR "Tibial" [tiab] OR "High" [tiab] OR "Open" [tiab], and "Closed or Closing" [tiab], and "Osteotomy" [MeSH] or "leg length" [tiab] or "limb length [tiab]". After the initial electronic search, relevant articles and bibliographies were searched manually. Articles identified were assessed individually for inclusion.

### Study selection

Study inclusion was decided independently by two reviewers, based on the predefined selection criteria. Titles and abstracts were read; if suitability could not be determined, the full article was evaluated. Studies were included in the meta-analysis if (1) they described preoperative and postoperative leg length in patients who underwent opening and closing wedge HTO; (2) they simultaneously reported direct comparisons of change in leg length for opening wedge and closing wedge HTO; (3) their primary outcomes included change in leg length after surgery; (4) they fully reported the sample numbers of their subjects in a final analysis, as well as means and standard deviations of leg length change; and (5) they used adequate statistical methods to compare the leg lengths before and after surgery.

### Data extraction

Two reviewers independently recorded data from each study using a predefined data extraction form. Any disagreement unresolved by discussion was reviewed by a third author.

Variables recorded included: (1) type of HTO–opening wedge, closing wedge, or both; (2) means and standard deviations of preoperative and postoperative leg length in open wedge and/or closed wedge HTO groups; and (3) sample size of each group. If these variables were not mentioned in the articles, we contacted the study authors by email to request the data.

### Assessment of methodological quality

Two reviewers independently assessed the methodological quality of each study using the Newcastle-Ottawa Scale,[15] as recommended by the Cochrane Non-Randomized Studies Methods Working Group. For our purposes, the Newcastle-Ottawa Scale’s star system, which awards stars depending on level of bias, was modified to include only low (one star), high, and unclear bias. Each study was judged on three criteria: the selection of the study groups, the comparability of the groups, and the ascertainment of either the exposure or outcome of interest for case-control or cohort studies. Any unresolved disagreements between reviewers were resolved by consensus or by consultation with a third investigator.

### Statistical analysis

The main outcomes of the meta-analysis were the mean difference in leg length change before and after surgery in either open wedge or closed wedge HTO. The mean difference and 95% confidence interval (CI) were used for continuous outcomes in comparing preoperative and postoperative leg length for each osteotomy. Heterogeneity was determined by estimating the proportion of between-study inconsistencies due to actual differences between studies, rather than differences due to random error or chance, using the I^2^ statistic, with values of 25%, 50%, and 75% considered low, moderate, and high, respectively. All statistical analyses were performed using RevMan version 5.2.

## Results

### Identification of studies

**Fig 1:**
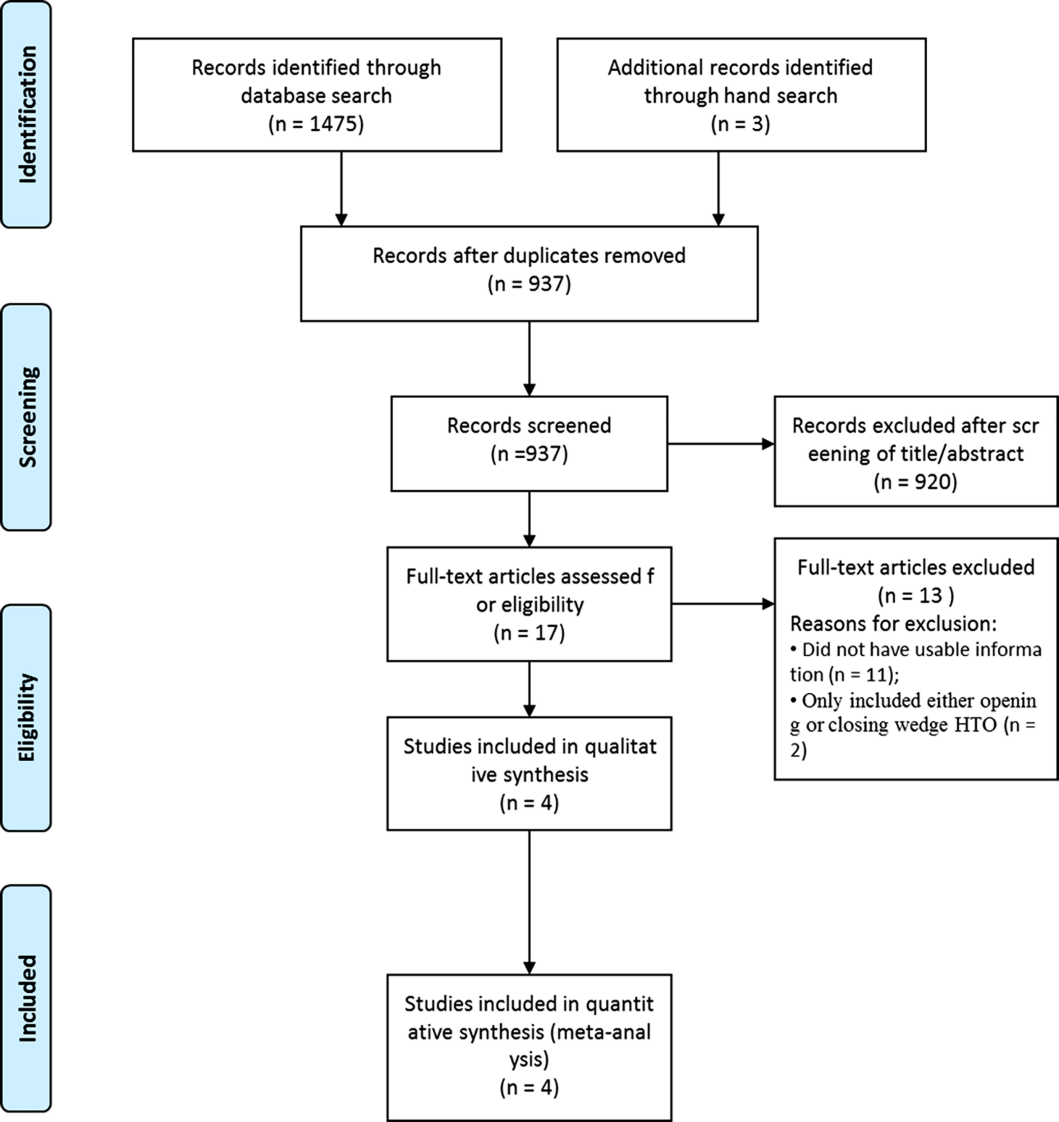
PRISMA (Preferred Reporting Items for Systematic Reviews and Meta-analyses) flow diagram of the identification and selection of the studies included in this meta-analysis.

Fig 1 shows the details of study identification, inclusion, and exclusion. An electronic search yielded 379 studies in PubMed (MEDLINE), 269 in EMBASE, 322 in Web of Science, 480 in SCOPUS, and 25 in the Cochrane Library. Three additional publications were identified through manual searching. After removing 541 duplicates, 937 studies remained; of these, 920 were excluded based on reading of the abstracts and full-text articles, and an additional 17 studies were excluded since they did not have usable information or reported the leg length change for only either open wedge or closed wedge HTO. After applying these criteria, four studies[11–14] were finally included in this meta-analysis.

### Study characteristics and quality of the included studies

All four included studies directly compared the leg length change in subjects before and after surgery between opening wedge and closing wedge HTO, with four studies also comparing flexion and extension moments before and after surgery. Of four included studies, two were retrospective comparison studies, and two were prospective comparison studies. All included studies showed a low risk of selection bias. All provided detailed demographic data of each open wedge and closed wedge HTO group. None assessed possible confounding factors. Of these four studies, three were considered high quality, with >5 points on the Newcastle-Ottawa Scale (Table 1).

**Table 1:** Characteristics and quality of studies included in the meta-analysis.

Author	Year	Study Type	Sample	size	Measured Parameters	Time point of postoperative radiography	Quality score
			OW HTO	CW HTO			
Bae et al.[11]	2013	PCS	28	74	Pre-and Postoperative leg length	2 weeks	5
Kim et al.[12]	2016	PCS	30	30	Pre-and Postoperative leg length	1 year	6
Magnussen et al.[13]	2011	RCS	32	32	Pre-and Postoperative leg length	1 year	8
Nerhus et al.[14]	2015	RCS	35	35	Pre-and Postoperative leg length	6 months	6

PCS, prospective comparison study; RCS, retrospective comparison study; ADM, adduction moment; FLM, flexion moment; EXM

### Leg length change after opening-and closing wedge HTO

**Fig 2:**
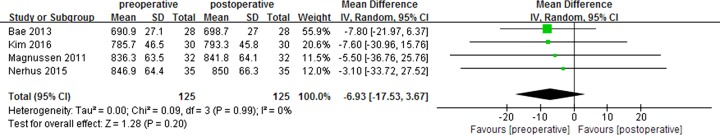
Forest plot showing changes in pre- and postoperative leg length in opening wedge high tibial osteotomy.

**Fig 3:**
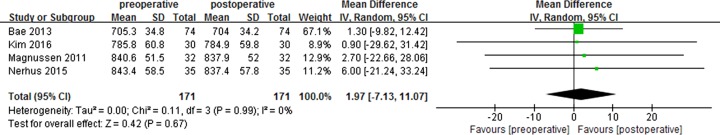
Forest plot showing changes in pre- and postoperative leg length in closing wedge high tibial osteotomy.

The four included studies evaluated a total of 125 and 171 knees that underwent opening and closing wedge HTO, respectively. The pooled mean leg length changes from before to after opening and closing wedge HTO were –6.93 mm (95% CI: –17.53 to 3.67 mm; P = 0.20; I^2^ = 0%, Fig 2) and 1.97 mm (95% CI: –7.13 to 11.07 mm; P = 0.67; I^2^ = 0%, Fig 3), respectively. These results seem to indicate that the leg length increased 6.93 mm after opening wedge and decreased 1.97 mm after closing wedge HTO. However, these leg length changes from before to after surgery were statistically not significant.

### Differences in leg length change between opening and closing wedge HTO

**Fig 4:**
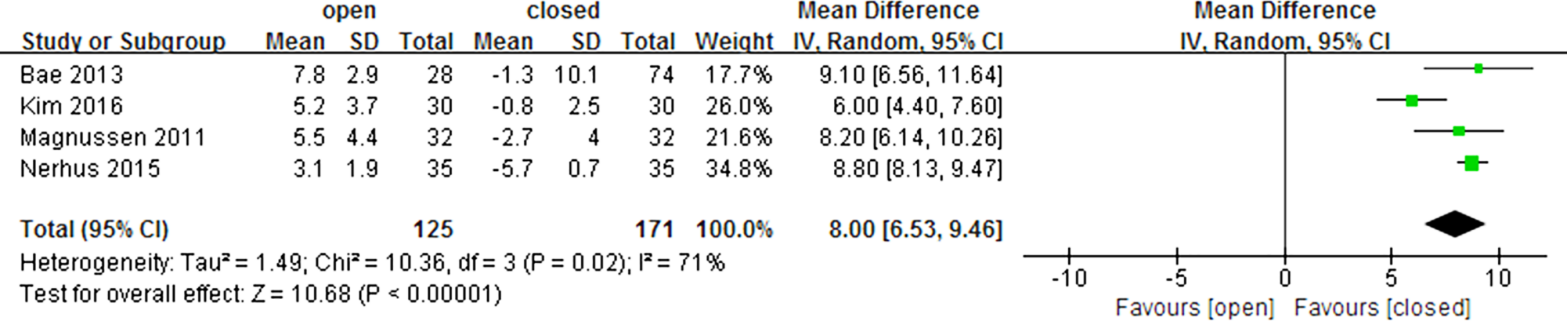
Forest plot showing the difference in leg length change between opening and closing wedge high tibial osteotomy.

All four included studies directly compared the leg length change from before to after surgery for opening and closing wedge HTO. The pooled mean difference in leg length change from before to after surgery in opening and closing wedge HTO was 8 mm (95% CI: 6.53 to 9.46 mm; P<0.001; I^2^ = 71%, Fig 4), indicating that leg length was 8 mm greater after opening wedge than after closing wedge HTO.

### Meta-regression analyses

The results of meta-regression analyses are reported in Table 2. The time point at which the postoperative radiograph was taken was not significantly associated with mean difference in leg length change from before to after surgery in opening and closing wedge HTO. This finding indicated that the difference in leg length change between opening-and closing wedge HTO was not influenced by the time point at which radiographs were taken after surgery.

**Table 2:** Meta-regression analyses of time of radiography after surgery and difference in mean leg length change from before to after surgery for opening and closing wedge high tibial osteotomy.

Variable	Coefficient	Standard error	P value	95% confidence interval
Change in leg length from before to after surgery				
Time of postoperative radiography	-0.056	0.034	0.243	‒0.205 to 0.092

## Discussion

The most important finding of this study was that leg length did not differ significantly between before and after surgery for both opening and closing wedge HTO. However, the difference in leg length change from before to after surgery was 8 mm greater in opening wedge than in closing wedge HTO.

The results of our current meta-analysis indicate that the change in limb length after either opening or closing wedge HTO was not statistically significant, and hence that a change in overall limb length does not seem to be a disadvantage of HTO. However, as this meta-analysis included only a small number of samples across four studies, the present results will need to be confirmed in an analysis of a greater number of cases. Theoretically, there was a trend toward increasing leg length after opening wedge HTO and decreasing leg length after closing wedge HTO. These trends were partially supported by our meta-analysis, because the pooled results of leg length change from before to after surgery showed 0 heterogeneity. In addition, all included opening wedge HTO studies showed increasing leg length after surgery, and all included closing wedge HTO studies showed decreasing leg length after surgery, although the pooled results of these data were not statistically significant. However, it is widely accepted that the magnitude of leg length change after surgery is greater in opening wedge than in closing wedge HTO, because leg lengthening effect induced by valgus realignment could amplify the leg length increment in opening wedge HTO, but could offset the leg length decrement in closing wedge HTO.[12] In the current meta-analysis, the amount of leg length increment was 6 mm in opening wedge HTO and the leg length decrement was 2 mm in closing wedge HTO, although these values were not statistically significant.

The statistical insignificance of leg length change from before to after opening and closing wedge HTO may reflect the small magnitude of leg length change after surgery rather than mathematically calculated amounts. In closing wedge HTO, as described above, the valgus realignment effect could offset the tibial metaphyseal bone loss. Therefore, if valgus realignment effects are greater than tibial metaphyseal bone loss, the net leg length change could increase. Even in opening wedge HTO, the shifting weight-bearing force from medial to lateral compartment could offset the effect of tibial lengthening after opening wedge HTO,[11] especially in cases with severe soft tissue laxity on the lateral side, because weight-bearing force can lead to overcorrection of alignment despite preoperative planning, which can shorten the leg length more than required for adequate correction. In addition, the increment of posterior slope after opening wedge HTO reduces the leg lengthening effect of opening wedge HTO, because the increased posterior slope leads to anterior translation of the tibia,[16] which could decrease the magnitude of leg lengthening effect in open wedge HTO.

There was no general consensus about the extent to which the change in leg length could result in clinically relevant problems such as back pain and progression of knee osteoarthritis due to alteration of gait kinematics.[17] Some degree (less than 2 cm) of leg length discrepancy can be allowed in humans because of a compensatory mechanism that dynamically lengthens the shorter limb and shortens the longer limb,[18] probably to minimize the displacement of the body center of mass and consequently reduce body energy expenditure.[19] However, a leg length discrepancy–whether lengthening or shortening–should be avoided as much as possible, because previous studies demonstrated that leg lengthening, even by less than 1 cm, increases the chance of developing knee osteoarthritis in the longer limb,[20] and that correcting leg length discrepancy of only 5.6 mm relieved chronic low back pain, possibly by reducing lumbar spine lateral flexion and rotation.[21] The result of the current meta-analysis showed an apparent difference in leg length change between opening wedge and closing wedge HTO; the opening wedge HTO resulted in an 8-mm greater leg length than closing wedge HTO. This small difference, although less than 1 cm, could induce clinically important symptoms, according to the results of the above two studies. Therefore, orthopedic surgeons should carefully consider leg length changes when deciding the type of HTO. If patients have a shorter leg length on the operative side than on the contralateral side, open wedge HTO should be considered rather than closing wedge HTO. In contrast, closing wedge HTO rather than opening wedge HTO should be chosen for cases in which leg lengthening is anticipated after surgery. Moreover, when treating patients with bilateral medial osteoarthritic varus knees and equal leg lengths, the same type of HTO should be performed on both legs.

Several limitations should be taken into consideration when interpreting the results of the current study. There are other factors that could affect leg length change after HTO. The leg length change could be influenced by the amount of alignment correction. It has been reported in the literature that the greater the amount of correction of limb alignment, the greater the leg length, even after closing wedge HTO. In addition, radiographs taken at different time points after surgery could also affect leg length change, because some residual flexion contractures could remain for up to 6 months after surgery. Also, the leg length determined from radiographs may depend on the conditions used when recording the radiographs; however, all of the studies included in this meta-analysis considered the effect of radiographic magnification. About half of the studies included in this meta-analysis were also retrospective observational comparison studies. These were influenced by different factors such as alignment correction amount, variable radiography time points, differences in the conditions used when recording radiographs, and low levels of evidence for the study design, resulting in inherent heterogeneity due to uncontrolled bias. However, the results of the current study with regard to leg length change from before to after surgery showed absolutely no heterogeneity, as represented by “zero (0)” I^2^ values, for both opening wedge and closing wedge HTO, which showed the robust reliability of this meta-analysis. In addition, the results of meta-regression analysis in our study showed no correlation between differences in leg length change in opening-and closing wedge HTO according to time of radiography after surgery. Finally, this study did not evaluate the effects of leg length change in opening or closing wedge HTO on clinical results such as knee osteoarthritis and back pain due to spine problems. Future research should examine these effects.

## Conclusions

Leg length did not change significantly from before to after surgery in both opening- and closing wedge HTO. However, leg length change from before to after surgery was 8 mm greater in opening wedge HTO than in closing wedge HTO.

## Supporting information

S1 PRISMA Checklist (PDF)

S1 Search Strategy (DOCX)
