# Supplementary material for: Leg length change after opening wedge and closing wedge high tibial osteotomy: A meta-analysis
Source: PLoS One. 2017 Jul 13;12(7):e0181328. doi: 10.1371/journal.pone.0181328 (PMC5509317; doi:10.1371/journal.pone.0181328)
Supplement: S1 Search Strategy — (DOCX) [file pone.0181328.s002.docx]

MEDLINE

1. Knee[TIAB] OR Knees[TIAB] OR medial[tiab] OR "Tibia"[tiab] OR Tibias[tiab] OR Tibial[tiab] OR media[tiab] OR tibiae[tiab] 475377
2. "Knee Joint"[Mesh] OR "Tibia"[Mesh] 75461
3. 1 OR 2 493137
4. Osteoarthritides[tiab] OR Osteoarthritis[tiab] OR Osteoarthrosis[tiab] OR Osteoarthroses[tiab] OR gonarthrosis[tiab] OR gonarthroses[tiab] 50368
5. 4 AND 3 22094
6. "Osteoarthritis, Knee"[Mesh] 13577
7. 6 OR 5 26329
8. "Osteotomy"[Mesh] 28874
9. “high tibial osteotomy”[tiab] OR “high tibial osteotomies”[tiab] 1074
10. 9 OR 8 29187
11. "Postoperative Complications"[Mesh:NoExp]
12. "Lower Extremity"[Mesh] 146357
13. lengths[tiab] OR length[tiab] OR height[tiab] OR “Lower Extremity”[tiab] OR “Lower Extremities”[tiab] OR “Lower Limb”[tiab] OR “Lower Limbs”[tiab] 708661
14. 10-13/OR 1010296
15. 5 AND 10 AND 14 412
16. 15 NOT (review[Publication Type] OR "review literature as topic"[MeSH Terms]) 379

EMBASE

1. Knee:ab,ti OR Knees:ab,ti OR medial:ab,ti OR "Tibia":ab,ti OR Tibias:ab,ti OR Tibial:ab,ti OR media:ab,ti OR tibiae:ab,ti 740527
2. 'knee'/exp OR 'tibia'/exp 87410
3. 1 OR 2 756001
4. Osteoarthritides:ab,ti OR Osteoarthritis:ab,ti OR Osteoarthrosis:ab,ti OR Osteoarthroses:ab,ti OR gonarthrosis:ab,ti OR gonarthroses:ab,ti 68017
5. 4 AND 3 31413
6. 'knee osteoarthritis'/exp 22560
7. 6 OR 5 **37741**
8. 'osteotomy'/exp 37192
9. “high tibial osteotomy”:ab,ti OR “high tibial osteotomies”:ab,ti 1301
10. 8 OR 9 **43104**
11. 'bone length'/exp OR 'leg lengthening'/exp 3717
12. lengths:ab,ti OR length:ab,ti OR height:ab,ti OR 'lower extremity':ab,ti OR 'lower extremities':ab,ti OR 'lower limb':ab,ti OR 'lower limbs':ab,ti 856669
13. 10 OR 12 858543
14. 5 AND 10 AND 13 291
15. 14 NOT reviews/it 269

COCHRANE

1. Knee OR Knees OR medial OR Tibia OR Tibias OR Tibial OR media OR tibiae :ab,ti,kw 35605
2. MeSH descriptor: [Knee Joint] explode all trees 2794
3. MeSH descriptor: [Tibia] explode all trees 484
4. 1 OR 2 OR 3 35608
5. Osteoarthritides OR Osteoarthritis OR Osteoarthrosis OR Osteoarthroses OR gonarthrosis OR gonarthroses :ab,ti,kw 8382
6. 4 AND 5 5238
7. MeSH descriptor: [Osteoarthritis, Knee] explode all trees 2234
8. 6 OR 7 **5238**
9. MeSH descriptor: [osteotomy] explode all trees 632
10. osteotomy OR osteotomies:ab,ti,kw 1106
11. 9 OR 10 **1141**
12. MeSH descriptor: [Postoperative Complications] this term only 16039
13. MeSH descriptor: [Lower Extremity] explode all trees 6119
14. lengths OR length OR height OR “Lower Extremity” OR “Lower Extremities” OR “Lower Limb” OR “Lower Limbs”:ab,ti,kw
15. 12-14/OR 59293
16. 5 AND 11 AND 15 29
17. 16/trials 25

Web of Science

1. TOPIC: (Knee OR Knees OR medial OR Tibia OR Tibias OR Tibial OR media OR tibiae) OR TITLE: (Knee OR Knees OR medial OR Tibia OR Tibias OR Tibial OR media OR tibiae) 1326927
2. TOPIC: (Osteoarthritides OR Osteoarthritis OR Osteoarthrosis OR Osteoarthroses OR gonarthrosis OR gonarthroses) OR TITLE: (Osteoarthritides OR Osteoarthritis OR Osteoarthrosis OR Osteoarthroses OR gonarthrosis OR gonarthroses) 63697
3. 1 AND 2 32524
4. TOPIC: (“osteotomy” OR “osteotomies”) OR TITLE: (“osteotomy” OR “osteotomies”) 22954
5. TOPIC: (lengths OR length OR height OR “Lower Extremity” OR “Lower Extremities” OR “Lower Limb” OR “Lower Limbs”) OR TITLE: (lengths OR length OR height OR “Lower Extremity” OR “Lower Extremities” OR “Lower Limb” OR “Lower Limbs”) 1267369
6. 3 AND 4 AND 5 340
7. 6 Refined by: [excluding] DOCUMENT TYPES: ( REVIEW ) 322

SCOPUS

1. TITLE-ABS(Knee OR Knees OR medial OR Tibia OR Tibias OR Tibial OR media OR tibiae ) 2606048
2. INDEXTERMS("Knee Joint" OR "Tibia") 92295
3. 1 OR 2 2628764
4. TITLE-ABS(Osteoarthritides OR Osteoarthritis OR Osteoarthrosis OR Osteoarthroses OR gonarthrosis OR gonarthroses) 59511
5. 4 AND 3 26619
6. INDEXTERMS("Osteoarthritis, Knee") 12877
7. 6 OR 5 **30720**
8. (INDEXTERMS("Osteotomy")) OR (TITLE-ABS(“osteotomy” OR “osteotomies”)) **47747**
9. (INDEXTERMS("Postoperative Complications" OR "Lower Extremity")) OR (TITLE-ABS(lengths OR length OR height OR “Lower Extremity” OR “Lower Extremities” OR “Lower Limb” OR “Lower Limbs”)) 2375288
10. 7 AND 8 AND 9 549
11. 10 AND ( EXCLUDE ( DOCTYPE , "re" ) ) 480

| MEDLINE | 379 |
| --- | --- |
| EMBASE | 269 |
| COCHRANE | 25 |
| WOS | 322 |
| SCOPUS | 480 |
| TOTAL | 1475 |
| DUPLICATE | 541 |
| FINAL | 934 |
